# Retention of Primary Bile Acids by Lupin Cell Wall Polysaccharides Under In Vitro Digestion Conditions

**DOI:** 10.3390/nu11092117

**Published:** 2019-09-05

**Authors:** Susanne Naumann, Ute Schweiggert-Weisz, Dirk Haller, Peter Eisner

**Affiliations:** 1 ZIEL-Institute for Food & Health, TUM School of Life Sciences Weihenstephan, Technical University of Munich, 85354 Freising, Germany (D.H.) (P.E.); 2 Fraunhofer Institute for Process Engineering and Packaging (IVV), 85354 Freising, Germany; 3 Chair of Nutrition and Immunology, TUM School of Life Sciences Weihenstephan, Technical University of Munich, 85354 Freising, Germany

**Keywords:** cholesterol, dietary fibre, bile acid binding, bile acid excretion, viscosity, viscoelastic properties, lignin, cellulose

## Abstract

Interference of dietary fibres with the enterohepatic circulation of bile acids is proposed as a mechanism for lowering cholesterol. We investigated how lupin hull and cotyledon dietary fibres interact with primary bile acids using an in vitro model under simulated upper gastrointestinal conditions. Cell wall polysaccharides were isolated and extracted to separate pectin-like, hemicellulosic, and lignocellulosic structures. Lupin hull consisted mainly of structural components rich in cellulose. The viscosity of the in vitro digesta of lupin hull was low, showing predominantly liquid-like viscoelastic properties. On the other hand, lupin cotyledon fibre retarded bile acid release due to increased viscosity of the in vitro digesta, which was linked with high contents of pectic polymers forming an entangled network. Molecular interactions with bile acids were not measured for the hull but for the cotyledon, as follows: A total of 1.29 µmol/100 mg DM of chenodesoxycholic acids were adsorbed. Molecular interactions of cholic and chenodesoxycholic acids were evident for lignin reference material but did not account for the adsorption of the lupin cotyledon. Furthermore, none of the isolated and fractionated cell wall materials showed a significant adsorptive capacity, thus disproving a major role of lupin cell wall polysaccharides in bile acid adsorption.

## 1. Introduction

As a sustainable alternative to soybean products, interest in the inclusion of sweet lupins in food is growing [1]. Next to their large proportions of plant protein, lupin seeds are a fibre source which has received little attention up to now [2]. Dietary fibres are mainly plant cell wall polysaccharides, such as celluloses, hemicelluloses, and pectin. They are characterized by their resistance to enzymatic degradation in the upper gastrointestinal tract, varying in their structural and nutritional properties [3,4]. Lupin kernel fibres—in contrast to insoluble cereal fibres—have a smooth texture, a neutral taste, and a white colour. This makes lupin kernel fibres an excellent non-intrusive ingredient for fibre enrichment, which shows high palatability and sensory acceptability in dietary interventions [5].

Legume seed fibres are derived from two main tissues, as follows: The hull (seed coat) and the cotyledons (kernels), which are therefore often referred to as outer and inner fibre. Legume hulls are rich in fibre, contain high amounts of cellulose and insoluble hemicelluloses and vary in lignin contents. Cotyledons contain less dietary fibre and are poorer in structural components (cellulose and lignin). A special feature of lupin cotyledons is the storage of high amounts of non-cellulose non-starch polysaccharides in their cell walls [6]. Dietary fibre contents of lupin cotyledons range considerably, between 11.4 and 40.1 g/100 g dry matter (DM), depending on genetic and environmental effects [7,8].

There is clear evidence that a diet rich in dietary fibre provides protection against diet-related disorders such as obesity, type 2 diabetes, cardiovascular disease, and colon cancer [9,10]. Accordingly, health benefits of lupin kernel fibre have been demonstrated in several in vivo studies. The consumption of lupin kernel fibre was associated with a decrease of blood cholesterol levels, lowering of blood pressure, improvement of insulin sensitivity, and favourable alteration of the gut microbiome [11,12,13,14].

Our recent in vitro findings suggest that the interaction of lupin kernel fibres with primary bile acids may contribute to the cholesterol-lowering properties of lupins [15]. Primary bile acids, tauro- and glycoconjugated cholic, and chenodesoxycholic acids, are synthesized in the liver from cholesterol and act as emulsifiers in the small intestine, enabling fat digestion and absorption. Bile acid reabsorption is mainly achieved by active transport in the terminal ileum [16]. Dietary fibres may interact with bile acids, withdrawing them from the enterohepatic circulation. By this means endogenous bile acid synthesis is stimulated, which could explain the cholesterol-lowering effect of dietary fibres [17]. Responsible components for decreased re-uptake of bile acids in the ileum and mechanisms of bile acid retention remain to be fully elucidated.

Interactions of dietary fibre with bile acids can be differentiated in adsorptive effects and the entrapment of bile acids in viscous chyme matrices using an in vitro model based on in vitro digestion, dialysis and kinetic analysis [18]. For a lupin kernel fibre preparation (with a dietary fibre content of 83.4 g/100 g DM), a retardation of bile acid release of about 50% due to increased viscosity of the digesta was observed for all primary bile acids. While cholic acids were not significantly adsorbed, an adsorption of 1.74 µmol/100 mg DM was reported for chenodesoxycholic acids [15,18]. However, it is unclear which lupin fibre fractions contribute to these interactions. Increasing the viscosity during digestion is mainly attributed to water-soluble fibres, which impede the diffusion and absorption of bile acids [19]. Although lupin kernel fibre is described as primarily insoluble, the increased viscosity under simulated digestion may be due to pectic substances with rheological properties similar to pectin [20].

While there is a broad agreement on the contribution of dietary fibre to viscosity formation, data regarding adsorptive capacities of individual dietary fibre structures are contradictory. As recently reviewed by Singh et al. [21], bile acid binding capacities were reported for numerous dietary fibre sources during the last decades. However, the term bile acid binding is regularly chosen by authors without considering the underlying mechanism, which impedes the differentiation of adsorptive and viscous effects [18]. On the other hand, some studies reported bindings, which were distinctly independent of viscosity due to thermal or enzymatic treatment of dietary fibres, e.g., as described for oat [22,23]. Since no direct molecular interaction with bile acids could be determined for β-glucan [24], the soluble fraction of oat, adsorption could be related to insoluble dietary fibre structures [25]. Analogously, adsorption by insoluble dietary fibres could account for the bile acid adsorption revealed for lupin fibre [15]. However, further studies are needed to identify the relevant structures and to characterize the adsorptive mechanism.

In our previous study, we positively correlated bile acid adsorption with bile acid hydrophobicity, which indicates an underlying mechanism based on hydrophobic interactions [15]. The main hydrophobic component of fibre is lignin, which occurs predominantly in structural plant components. Lignin could be introduced to lupin kernel fibre preparations through remaining hulls after the technical de-hulling processes [26]. Lignin has long been considered as the responsible component for bile acid adsorption [27,28,29,30,31]. However, the major role of lignin in bile acid adsorption was questioned by other studies [32,33,34,35]. It is thus not entirely understood whether lignin adds to molecular interactions of lupin fibre with bile acids.

We hypothesize that bile acid retardation by dietary fibres is a synergic effect of viscosity formation and hydrophobic interactions of dietary fibres with bile acids [15,17,18,19,36]. It is essential to gain detailed knowledge of the plant tissue, processing of fibre preparations, and their physiochemical properties to understand the underlying relationships between cell wall polymer structures and their functional effects on bile acid metabolism.

The aim of this study was to evaluate whether and to what extent lupin dietary fibres contribute to the viscous and adsorptive interactions with primary bile acids. Cell wall components were isolated from lupin cotyledon and hull and sequentially extracted to separate pectin-like hemicellulosic and lignocellulosic structures. Cellulose and lignin were used as references for bile acid interaction studies. The assessment of in vitro bile acid interactions was combined with rheological and dietary fibre characterisations to obtain a profound knowledge about the fractions of the fibre responsible for interactions with specific bile acids.

## 2. Materials and Methods

### 2.1. Chemicals and Enzyme Preparations

Cellulose VITACEL^®^ (L 600-30) was supplied by J. Rettenmaier & Soehne GmbH & Co. KG (Rosenberg, Germany). Taurocholic acid sodium salt hydrate (CAS 345909-26-4), sodium taurochenodeoxycholate (CAS 6009-98-9), sodium glycocholate hydrate (CAS 338950-81-5), sodium glycochenodeoxycholate (CAS 16564-43-5), PRONASE^®^ (53702, Protease, Streptomyces griseus, CAS 9036-06-0), lignin alkali (CAS 8068-05-1), L-α-lecithin (Egg Yolk, Highly Purified-CAS 8002-43-5), pancreatin from porcine pancreas (8 × USP specifications), and pepsin from porcine gastric mucosa (3200–4500 units/mg protein) were purchased from Merck KGaA (Darmstadt, Germany). All other reagents and chemicals used were of analytical grade and supplied by VWR (Darmstadt, Germany).

### 2.2. Raw Material and Pretreatment of Lupin Seeds

*L. angustifolius* L. Boregine (harvest 2016) were provided by Saatzucht Steinach GmbH & Co. KG (Steinach, Germany). The cleaned lupin seeds were de-hulled using an underrunner disc sheller (Streckel & Schrader KG, Hamburg, Germany). The cotyledons were separated from the hulls using a zigzag air-classifier (Hosokawa Alpine AG, Augsburg, Germany). Remaining hull material was removed manually and the germ was discarded. Subsequently, the kernels were flaked using a flaking mill (Streckel & Schrader KG, Hamburg, Germany). The obtained yellow flakes were de-oiled by Soxhlet extraction using n-hexane (‘white flakes’), which was evaporated in a cold air stream after oil removal. Hulls were sieved to remove adherent residues of the cotyledons. Finally, the hulls and de-oiled white flakes were milled using a Retsch ZM-200 mill (Duesseldorf, Germany) to fit through a 500 µm screen.

### 2.3. Chemical Composition

Dry-matter and ash contents were analysed on a thermogravimetric basis at 105 °C and 550 °C using a TGA 601 analyser (Leco, St. Joseph, MI, USA) following the official methods, L 18.00-12 and L 15.00-7, respectively [37]. Nitrogen (AOAC Official Method 968.06) was determined according to the Dumas combustion method using a Nitrogen Analyzer TruMac N (LECO Instrumente GmbH, Mönchengladbach, Germany) and protein was calculated as N × 5.8 [38]. Fat content was analysed following the method of Caviezel [39]. Determination of starch was carried out by glucose determination applying the hexokinase method described by Beutler [40]. Carbohydrates were calculated as 100− (ash + protein + fat + total dietary fibre).

### 2.4. Dietary Fibre Analyses

The soluble and insoluble dietary fibre content was determined on an enzymatic-gravimetric basis (AOAC Official Method 991.43, Association of Official Analytical Chemists [38]). Neutral detergent fibre (NDF), acid detergent fibre (ADF), and acid detergent lignin (ADL) were analysed following the method described by van Soest et al. [41].

### 2.5. Monosaccharide Composition

Cell walls were hydrolysed by Saeman procedure (Sulphuric acid hydrolysis: 1 h 12 M at 30 °C, followed by 3 h 1 M at 100 °C) and monosaccharides (arabinose, rhamnose, galactose, glucose, xylose, mannose) were determined by high performance anion exchange chromatography with pulsed amperometric detection. The total uronic acid was determined by spectrophotometric quantification [42].

### 2.6. Fractionation of Lupin Cotyledons and Hulls

**Figure 1:**
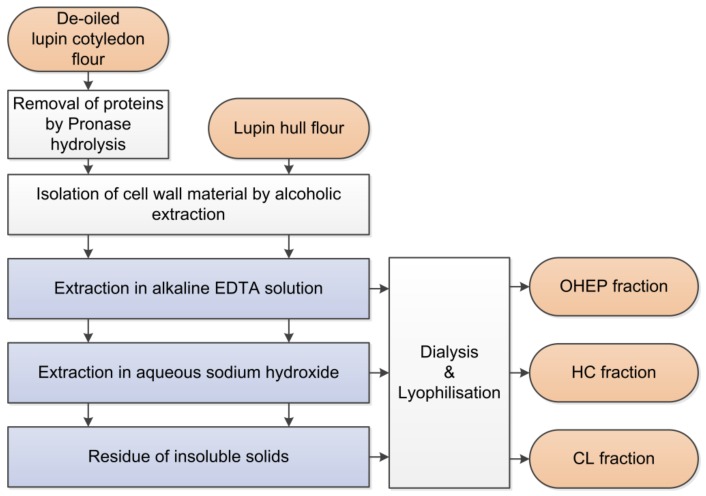
Sequential fractionation of cell wall material obtained from the cotyledon and the hull of *L. angustifolius* L. Boregine (pectin-like fraction (OHEP), hemicellulose fraction (HC), and cellulose-lignin fraction (CL)) [43,44,45].

Hull flour and de-oiled cotyledon flour were obtained as described in Section 2.2 and fractionated as illustrated in Figure 1. Protein in lupin cotyledons was removed by Pronase hydrolysis (Section 2.6.1). Subsequently, the cell wall material (CWM) was isolated from the residue of cotyledon hydrolysis and the hulls (Section 2.6.2). Sequential extraction of the CWM yielded three fractions, as follows: NaOH–EDTA-soluble pectin-like fraction (OHEP), hemicellulose fraction (HC), and cellulose-lignin fraction (CL) (Section 2.6.3) [43,44,45].

#### 2.6.1. Removal of Protein from Lupin Cotyledons by Pronase Hydrolysis

De-oiled lupin cotyledon flour (5% (*w/v*)) was homogenised in phosphate buffer (0.1 M, pH 7.5) using an Ultra-Turrax blender (IKA, Staufen, Germany). Pronase (5000 IU/100 g lupin flour) was added and incubated at 30 °C for 5 h, followed by centrifugation at 5000× *g* for 15 min. The residue was washed five times with excess water and recovered by centrifugation [45].

#### 2.6.2. Isolation of Cell Wall Material

The residue of cotyledon Pronase hydrolysis (Section 2.6.1) and the hull flour (Section 2.2) were suspended (10% (*w/v*)) and extracted in boiling ethanol (80% (v/v)) for 1 h under permanent mixing. The solid fraction was isolated from the alcohol phase by filtration using a Büchner funnel and blue ribbon filter paper. The extraction was repeated until the alcohol phase was clear, which was achieved after three repetitions for cotyledon residue and six repetitions for hull flour. The residue was stirred overnight in acetone and then filtered using a G3 glass sinter filter. The solids were transferred into a tared glass vessel and dried at 40 °C for 24 h. The yield of CWM was determined gravimetrically (*n* = 3) [44].

#### 2.6.3. Sequential Extraction of Cell Wall Material

The sequential extraction was adapted from Schieber et al. [43]. Extraction conditions were attained using a water bath equipped with a magnetic stirrer. Centrifugation (15,000 × *g*, 25 min) with subsequent filtration of the supernatant using a Büchner funnel and black ribbon filtration paper was used to separate the liquid phase from the residue, which was further extracted afterwards. Filtrates were pooled, concentrated using a rotary evaporator, dialysed (Servapor^®^ dialysis tubings 29 mm, MWCO 12–14 kDa, SERVA Electrophoresis GmbH, Heidelberg, Germany) for three days against demineralized water and lyophilized.

CWM (Section 2.6.2) was suspended (2% (*w/v*)) in alkaline EDTA solution (0.05 M NaOH; 0.5 mM EDTA) and extracted twice at 30 °C for 1h. After washing twice with demineralized water, the OHEP fraction was obtained. Aqueous sodium hydroxide (16% (*w/w*)) was used to extract the hemicelluloses (HC) at 30 °C for 5 h. The pellet was rinsed twice and the pH of the resulting fraction was adjusted to 6.5 using hydrocholic acid (37%). The residue was defined as cellulose-lignin fraction (CL). The proportions of the single fractions were determined gravimetrically by repeated fractionation of 1 g CWM (*n* = 3). In order to obtain material for further analyses, a larger extraction of 100 g CWM was additionally carried out.

### 2.7. In Vitro Digestion

Reference materials (cellulose and lignin) and lupin fractions were diluted with demineralized water to obtain a constant dietary fibre concentration of 5% in the final digestion mixture. The in vitro digestion was performed according to the harmonized INFOGEST protocol [46,47] with slight modifications, as follows: Oral phase was performed without added α-amylase due to the low content of starch in all samples and 0.04% of sodium azide was added to all electrolyte fluids to reach a concentration of 0.02% in the final digestion mixture to avoid microbial growth. To study interactions with primary bile acids, a mixture (1:1:1:1) containing taurocholic acid (TCA), glycocholic acid (GCA), taurochenodesoxycholic acid (TCDC), and glycochenodesoxycholic acid (GCDC) was used. A blank digestion was performed using the same concentration of simulated digestion fluids, lecithin, pepsin, pancreatin, and bile, but the sample was replaced with demineralized water.

### 2.8. In Vitro Interactions with Bile Acids

A total of 4 g of in vitro digesta (Section 2.7), with 10 mM of primary bile acid mixture, was dialysed (16 mm Servapor^®^ 12–14 kDa cut-off dialysis tubings (SERVA Electrophoresis GmbH, Heidelberg, Germany)) against 36 mL of phosphate buffer (50 mM, pH 7, 0.02% sodium azide) using a shaking water bath at 150 rpm and 37 °C. At 1, 2, 4, 8, 12, 24, and 48 h dialysis time, 100 μL-aliquots of dialysate were analysed for permeated bile acids by high performance liquid chromatography as described in detail in Naumann et al. [15]. Bile acid release kinetics were exploited applying non-linear regression using SigmaPlot^®^, version 12.5 (Systat Software Inc., San Jose, CA, USA).

### 2.9. Rheological Measurements

Viscoelastic and viscosity measurements were conducted using a rotational rheometer (Physica MCR 301, Anton Paar, Graz, Austria). Triple determinations were performed using a parallel plate geometry (diameter: 25 mm, shear gap: 1 mm) (PP25-SN23060, Anton Paar, Graz, Austria) at constant temperature of 37 ± 0.1 °C.

#### 2.9.1. Viscosity

Before starting the measurement, samples were sheared at a shear rate of 5 s^−1^ for 20 s and allowed to rest for 20 s. The viscosity η was monitored as a function of the shear rate, which was analysed in a logarithmic scale ranging from 1–1000 s^−1^.

#### 2.9.2. Dynamic Oscillatory Rheology

The linear viscoelastic range was determined using strain sweep tests prior to the analysis of the viscoelastic behaviour by frequency sweep tests. Strain sweep tests were performed at an angular frequency of 10 s^−1^ and a strain ramp in a logarithmic scale ranging from 0.01% to 100% deformation of the sample. Based on the results for linear viscoelastic ranges, a constant strain of 0.5% sample deformation was set for the frequency sweep tests. Frequency sweep tests were performed with a logarithmic decreasing angular frequency ranging from 100 to 0.001 s^−1^. Storage (G′) and loss modulus (G″) were calculated and used to evaluate the viscoelastic properties of the samples.

### 2.10. Statistical Analysis

The results were evaluated statistically using R version 3.2.4 (www.r-project.org). In all analyses, *p* ≤ 0.05 was considered significant. After testing for homogeneity of variance (Bartlett test) and normal distribution (Shapiro–Wilk test), one-way ANOVA with a post-hoc Tukey test was applied to separate significant means.

## 3. Results and Discussion

### 3.1. Chemical and Dietary Fibre Composition

**Figure 2:**
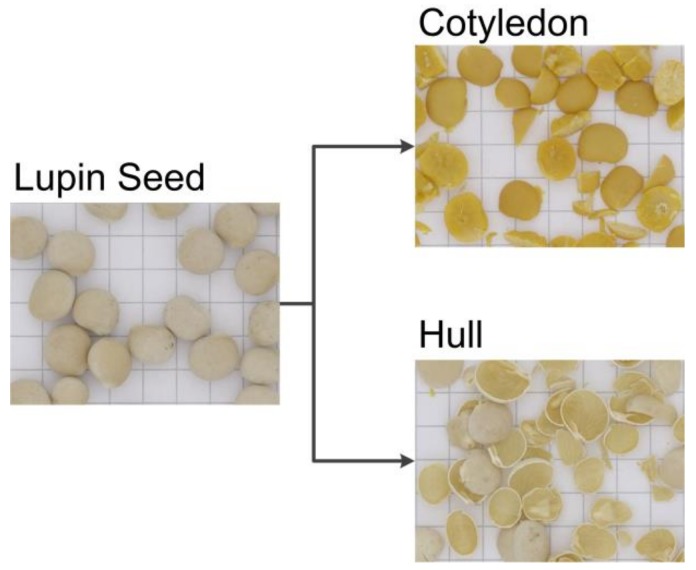
Seeds of *L. angustifolius* L. Boregine.

Lupin seeds of *L. angustifolius* L. Boregine (Figure 2) had an average weight of 189.7 ± 40.5 g/1000 seeds and dimensions of 7.3 mm × 6.2 mm × 5.3 mm and the hulls comprised 18.9 ± 2.4% of the total seeds (*n* = 10).

The chemical and dietary fibre compositions of the cotyledon and the hull of *L. angustifolius* L. Boregine are given in Table 1.

Protein (40.13 g/100 g DM), dietary fibre (37.77 g/100 g DM), and fat (9.47 g/100 g DM) represented the main components of lupin cotyledon; the compositional data was within the range reported in the literature [48,49,50]. Starch content of cotyledon was low (0.07 g/100 g DM), which was expected as starch is replaced by galactans as the reserve polysaccharide in lupin [51].

Dietary fibre composition detected for cotyledon and hull of *L. angustifolius* L. Boregine was comparable with values reported by Evans et al. [52] for three cultivars of the same lupin species. The cotyledon of *L. angustifolius* L. Boregine contained high amounts of dietary fibre (37.77 g/100 g DM), which was predominantly water-insoluble (34.59 g/100 g DM). Water-insoluble fibre determined by detergent-extraction (NDF) was 11.34 g/100 g DM. This underestimation in comparison to the enzymatic-gravimetric analysis of insoluble dietary fibre is probably due to a high content of water-insoluble pectin-like substances, which are lost during the NDF procedure [53]. Detergent extraction further revealed low levels of cellulose (ADF–ADL: 4.87 g/100 g DM) and lignin (ADL: 0.37 g/100 g DM). The low content of these structural components in the cotyledon was also reported by Evans et al. [52] and may be explained by the accumulation of non-structural material within the cell wall, which is presumed as a major polysaccharide reserve [54]. As expected, lupin hull mainly consisted of dietary fibre (91.31 g/100 g DM), which was predominantly insoluble (90.10 g/100 g DM) [6]. Hull fibre contained high amounts of cellulose (ADF–ADL: 72.87 g/100 g DM), while minor lignification was observed (1.95 g/100 g DM).

### 3.2. Fractionation and Monosaccharide Composition of Cell Wall Polysaccharides

**Figure 3:**
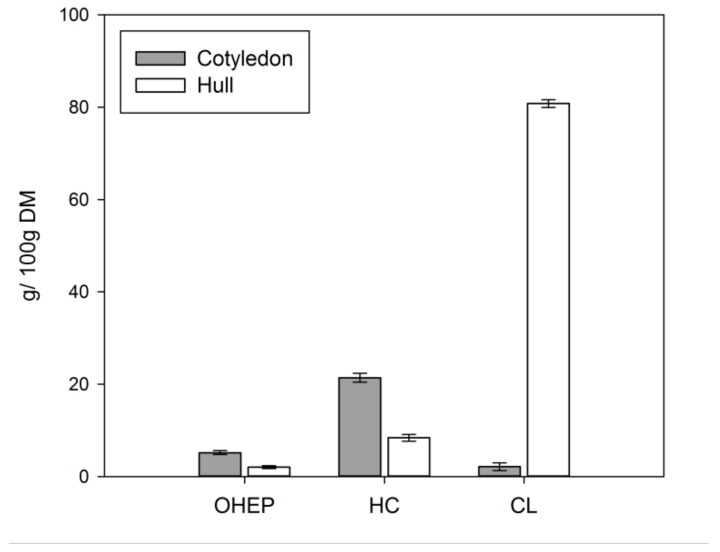
Yields of pectin-like fraction (OHEP), hemicellulose fraction (HC), and cellulose-lignin fraction (CL) isolated from the cotyledon and hull cell wall material of *L. angustifolius* L. Boregine (*n* = 3).

CWM constituted of 32.6 ± 0.3 g/100 g DM for lupin cotyledon and 93.1 ± 0.7 g/100 g DM for lupin hull. Recovery of the CWM after sequential extraction was 97.2 ± 1.4 % and 89.7 ± 0.5 % for cotyledon and hull, respectively. In a first step solubilisation of pectin-like substances from the isolated CWM was enhanced by EDTA in dilute alkaline solution (OHEP). Hemicelluloses were extracted with increasing alkali concentrations (HC), whereas the remaining alkali-insoluble residue predominantly consisted of cellulose and low amounts of lignin (CL) [43]. Sequential extraction yields for OHEP, HC, and CL fractions are given in Figure 3.

HC represented the main fraction of lupin cotyledon (21.4 g/100 g DM), followed by OHEP (5.2 g/100 g DM) and small amounts of CL (2.1 g/100 g DM). CL was predominant in lupin hull (80.8 g/100 g DM), while HC (8.4 g/100 g DM) and OHEP (2.0 g/100 g DM) was low. The monosaccharide composition of the cotyledon, hull and isolated pectin-like fraction (OHEP), hemicellulose fraction (HC), and cellulose-lignin fraction (CL) of *L. angustifolius* L. Boregine is given in Table 2.

The cell wall composition of the cotyledon (Table 2) is in agreement with previously reported results for *L. angustifolius* L. [8,51,52]. The main components were galactose (67.6%), arabinose (11.5%), and uronic acids (8.1%), with glucose (7.6%) and xylose (2.6%) being minor constituents. The glucose content is in agreement with the cellulose content obtained by detergent extraction (ADF–ADL: 4.87 g/100 g DM) and sequential extraction (Figure 3). The glucose and xylose contents further indicate the presence of xyloglucans or galacto-xyloglucans. The monosaccharide composition coincides with the study of Al-Kaisey and Wilkie [51], who described lupin cotyledon polysaccharides to comprise galactans, arabinogalactans, arabinans, rhamnogalacturonans, and galactoxyloglucans. For these galactans, Cheetham et al. [55] proposed a possible molecular structure. Thereafter, 1-4-linked long-chain galactans and highly branched 1-5-linked arabinans are linked to the rhamnosyl residues of a rhamnogalacturonan backbone. The galactose (71.8%) and rhamnose (1.7%) found in the HC fraction of the cotyledon indicated that this fraction partially consisted of pectic polymers that were not removed by the EDTA extraction. Higher yields for pectic fraction (71.0% and 61.4%) were described by Carre et al. [56] and Evans et al. [52] applying higher temperatures and lower pH during extraction with EDTA. Thus, although the yield was highest for HC, pectic polymers represented the main component of lupin cotyledon. The main components of the CL fraction were galactose (46.9%), glucose (24.8%), and arabinose (10.6%). While the glucose content is expected and accounts for cellulosic structures, the compositional data further indicates residual hemicellulose and pectic substances. This indicates that these components are strongly associated with the structural constituents of the cell wall.

As expected for legume seed hulls [6], the lupin hulls under study essentially contained cellulose, which accounts for the glucose present in the hull (59.1%) and the CL fraction (77.3%). The other main sugars were xylose (15.7%), uronic acids (12.1%), and arabinose (8.3%), which agrees with the data reported by previous authors for *L. angustifolius* L. and other lupin species [52,57]. This indicated that the hull, next to cellulose, mainly contained arabinoxylan hemicelluloses and pectic polysaccharides rich in uronic acids [52]. OHEP, which was the smallest hull fraction (2.0 g/100 g DM), showed a strikingly different composition, as follows: Uronic acids were predominant (29.7%), followed by mannose (25.9%), arabinose (20.7%), and galactose (13.6%). Besides pectic substances and arabinoxylan, this composition indicated the presence of galactomannans, which is considered a minor hull constituent [52]. The dominant sugar in HC was xylose (71.0%), which might originate from the secondary cell wall [57].

### 3.3. Rheological Measurements

#### 3.3.1. Viscosity

**Figure 4:**
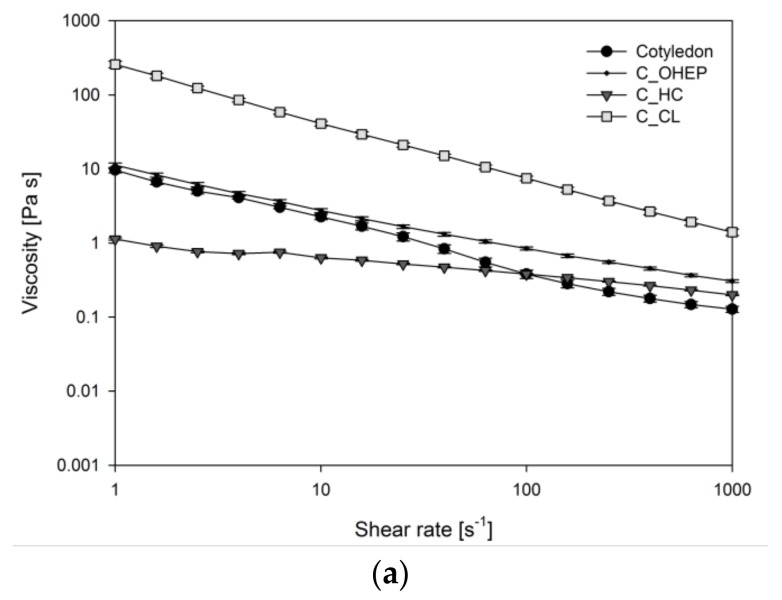

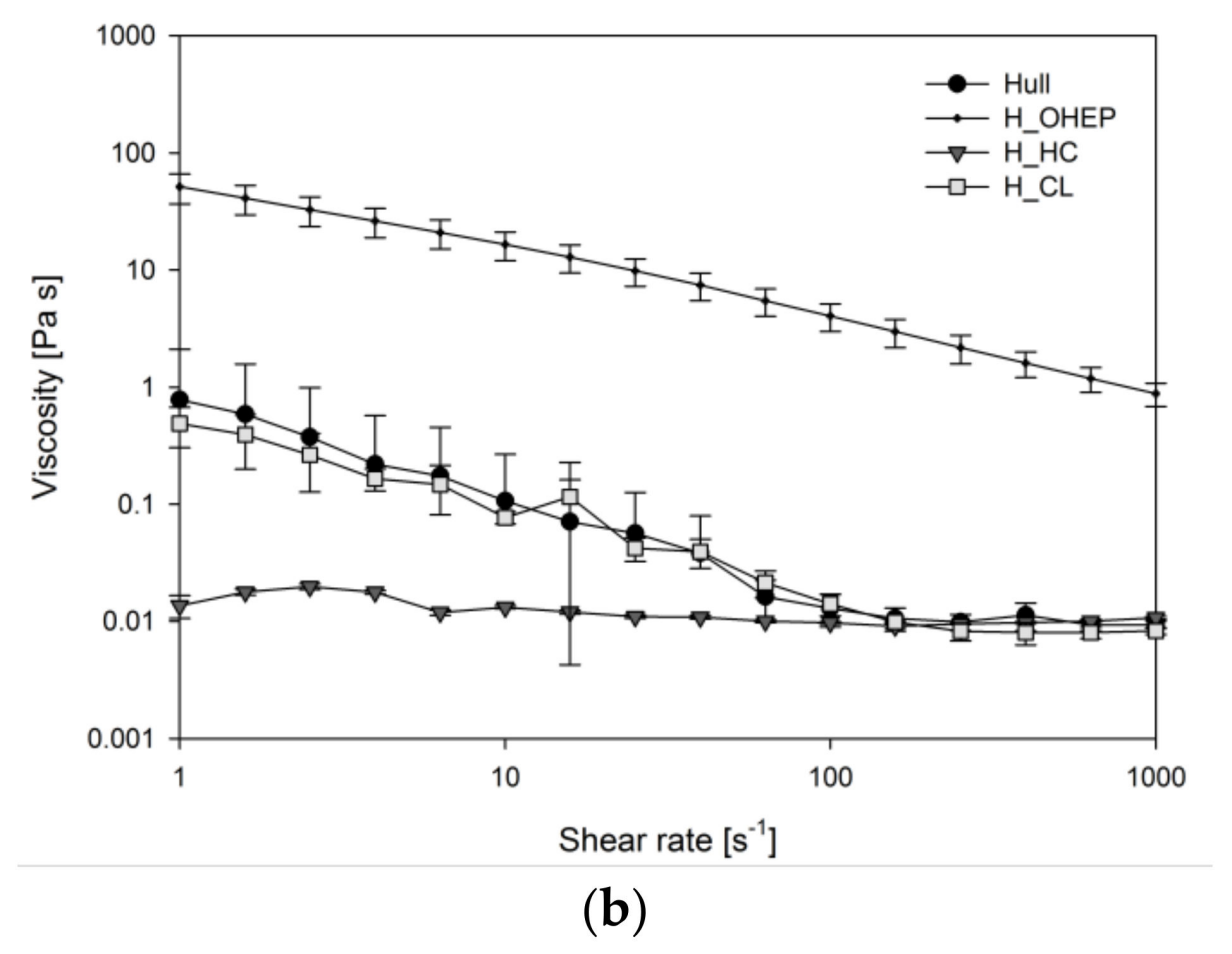
Viscosity of in vitro digesta containing fractionated dietary fibre (pectin-like fraction (OHEP), hemicellulose fraction (HC), cellulose-lignin fraction (CL)) derived from lupin cotyledon (**a**) and hull (**b**) of *L. angustifolius* L. Boregine (*n* = 3).

The viscosity of in vitro digested samples was tested for logarithmically increasing shear rates and which are reported in Figure 4.

The digested HC fractions of hull revealed nearly true viscous flow behaviour (viscosity independent of changes in shear rate). All other samples showed a similar pattern of shear thinning at high shear rates, which is typical for insoluble fibre suspensions. The flow curve of the digesta containing lupin hull and its CL fraction tended to a plateau value at high shear rates. This newton-like flow behaviour at high shear rates is typical of polymers without crosslinks or entanglements. As only low shear forces occur in the gastrointestinal tract, viscosity values at low shear rates (approximately 15 s^−1^) are most relevant with regard to bile acid release [36]. At low shear rates, flow curves of digesta containing lupin cotyledon and its OHEP fraction were almost identical (Figure 4a). The highest viscosity was observed for the CL fraction of the cotyledon, while HC showed the lowest viscosity at low shear rates. For digesta containing lupin hull and its dietary fibre fractions (Figure 4b), viscosity of OHEP was almost 2 decades higher than for hull and CL, which showed similar flow behaviour. As observed for cotyledon digesta, the HC fraction of hull showed the lowest viscosity. The viscosities of the in vitro digested reference materials cellulose and lignin were low (<0.02 Pa s at a shear rate of 15 s^−1^) and showed no significant deviation from blank digestion (*p* = 0.3).

#### 3.3.2. Dynamic Oscillatory Rheology

**Figure 5:**
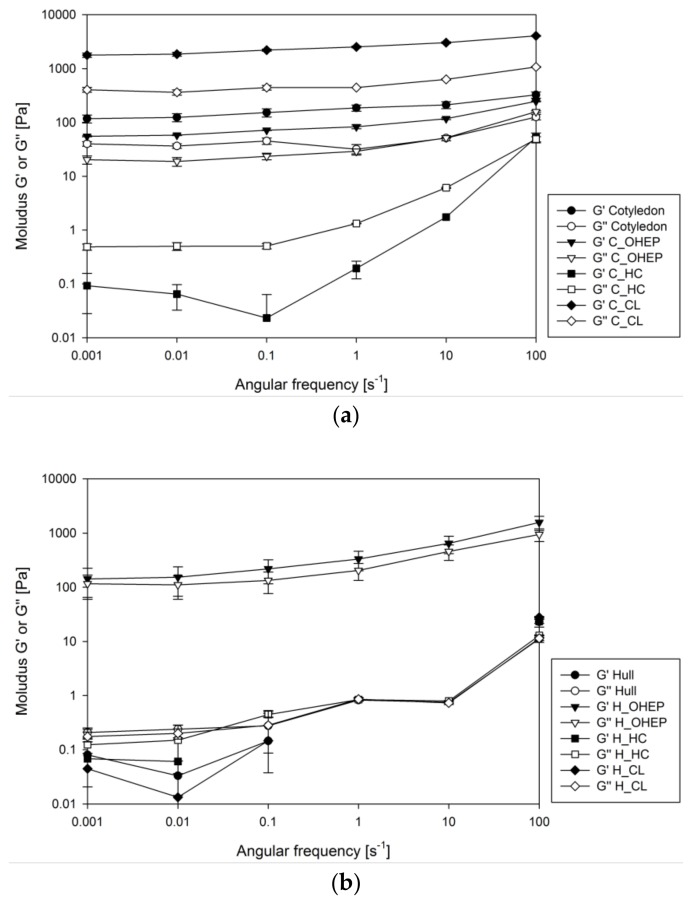
Frequency spectra of storage (G′) and loss (G″) moduli of in vitro digesta containing fractionated dietary fibre (pectin-like fraction (OHEP), hemicellulose fraction (HC), cellulose-lignin fraction (CL)) derived from lupin cotyledon (**a**) and hull (**b**) of *L. angustifolius* L. Boregine (*n* = 3).

The frequency spectra of storage (G′) and loss (G″) moduli of in vitro digesta containing fractionated dietary fibre derived from lupin cotyledon (a) and hull (b) are shown in Figure 5.

For the whole cotyledon as well as its OHEP and CL fraction (Figure 5a), both moduli were largely independent of frequency and a prevalence of G′ over G″ was observed, suggesting a highly crosslinked polymer solution or dispersion with high degree of structural integrity. For the HC fraction of the cotyledon, however, G″ was higher than G′ and the values of both G′ and G″ continuously decreased with decreasing frequencies towards a plateau at low frequencies. This indicates a weakly crosslinked polymer solution or dispersion with low structural strength. Similar viscoelastic properties were observed for the hull and its HC and CL fraction (Figure 5b). The OHEP fraction of the hull showed a small decrease of both moduli with continuous G′ > G″, signalling predominantly elastic properties.

#### 3.3.3. Evaluation of Rheological Properties

Although HC was the largest fibre fraction of lupin cotyledon (Figure 3), the rheological properties of the cotyledon digesta deviated strongly from this fraction. HC showed lower flow viscosity and predominantly liquid-like behaviour (G″ > G′). However, the viscosity and viscoelastic properties (predominantly solid-like behaviour (G′ > G″)) observed for the OHEP fraction was similar to the cotyledon. Therefore, we assume the rheology of the cotyledon to be associated with the polysaccharides extracted in the OHEP fraction. The rheological properties of the HC fraction indicate a low molecular weight and low crosslinking of the contained polymers. The rheological properties of the OHEP fraction, on the other hand, suggest a high molecular weight and high entanglement of the polymers. This is corroborated by the study of Carre et al. [56], who described a small EDTA soluble fraction of high molecular weight, which mainly consisted of galactose, as we also observed in this study. Due to its high cellulose content, the high viscosity and high structural integrity for the CL cotyledon fraction was not expected. The low viscosity of digesta containing cellulose was already described in previous studies [18]. We assume that the rheological properties of CL digesta can thus be ascribed to residual pectic polymers (Table 2). The higher structural strength, in comparison to the OHEP fraction, further suggests synergistic interactions between the pectic polymers and the structural lignocellulosic components.

As expected, the hull digesta showed rheological properties similar to the CL fraction, which was by far the largest hull fraction (Figure 3). In agreement to previous studies, the viscosity of the mostly cellulosic polymers was low [18]. Liquid-like behaviour was dominant in the frequency test, which is in line with the low water binding capacity of cellulose [58]. The OHEP fraction showed a high viscosity and high structural strength, indicating a high molecular weight and entanglement of the polymers. The properties of the hull HC were comparable to the HC fraction extracted from cotyledon. Due to the small proportions of OHEP and HC in the hull (Figure 3), no contribution of these fractions to the rheological behaviour of the hull was expected.

### 3.4. Interactions with Primary Bile Acids

Bile acid release kinetics were investigated for the primary bile acids glycocholic acid (GCA), taurocholic acid (TCA), glycochenodesoxycholic acid (GCDC), and taurochenodesoxycholic acid (TCDC). The concentration of primary bile acids after reaching equilibrium (C_f_) and the apparent permeability rate constant (k) were calculated following the first-order kinetic described in Equation (1) [36,59].

(1) $$C_{t}\;={\;C}_{f}\times \;\left(1-{e\;}^{-\;k\;t}\right)$$

Correlation coefficients for non-linear regression of bile acid release ranged between 0.977 and 0.999, which showed the high agreement of the kinetic fitting with the experimental data.

**Figure 6:**
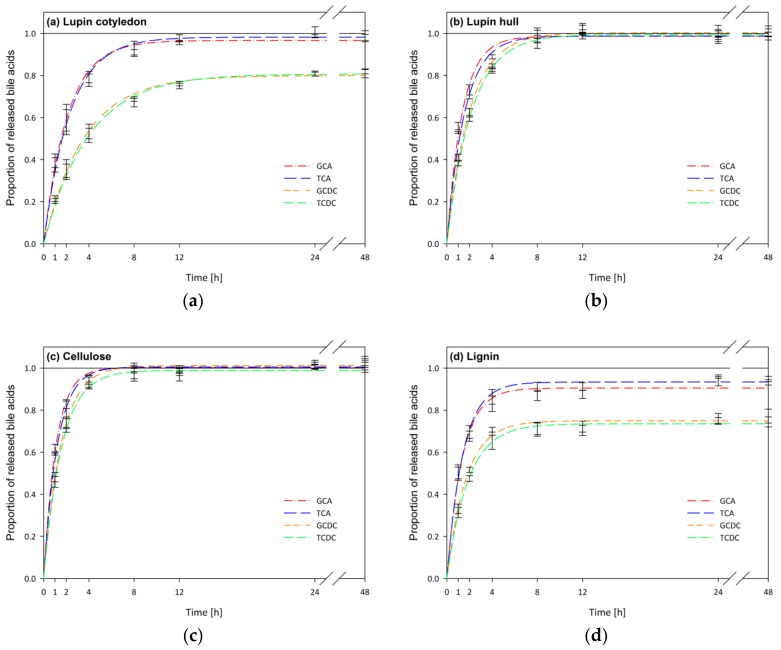
Diffusion kinetics of bile acid release (glycocholic acid (GCA), taurocholic acid (TCA), glycochenodesoxycholic acid (GCDC), and taurochenodesoxycholic acid (TCDC)) of in vitro digested cotyledon (**a**) and hull (**b**) of *L. angustifolius* L. Boregine, cellulose (**c**) and lignin (**d**) (*n* = 3).

Figure 6 shows the bile acid release kinetics for in vitro digesta containing lupin cotyledon (a) and hull (b) compared to the reference materials cellulose (c) and lignin (d).

If bile acid release studies are combined with rheological investigations after in vitro digestion, viscous and adsorptive interactions of samples with specific bile acid species can be differentiated by the parameters of the kinetic analysis [18]. The effects of the samples on the viscosity after in vitro digestion can be understood using rheological measurements (Section 3.3). Subsequently, the influence of the viscosity increase on specific bile acid species can be evaluated by comparing their apparent permeability rates, k (Equation (1)), which is further addressed in Section 3.4.1.

In case that bile acids are adsorbed to sample components, less bile acids are available for diffusion, resulting in a decrease of the equilibrium concentration, C_f_ (Equation (1)), in comparison to the blank digestion. In Figure 6 this incomplete release of bile acids is evident for lupin cotyledon (a) and lignin (d), which is examined and discussed in more detail in Section 3.4.2.

#### 3.4.1. Viscous Interactions

Apparent permeability rates (k) of fractionated CWM derived from lupin cotyledon and hull and the references, cellulose and lignin, are given in Table 3.

The apparent permeability rate constants described in Table 3 differed depending on the conjugation and hydroxylation of the primary bile acids (two-way ANOVA, *p* < 0.001). Release rates decreased following the order GCA > TCA > GCDC > TCDC, which can be explained by the micellar properties (critical micelle concentrations) of the different bile acids, as described in detail in Naumann et al. [15].

In vitro digested cotyledon and its dietary fibre fractions showed a retarded release of primary bile acids in comparison to the blank digestion (Table 3). The extent of the retardation was comparable to lupin fibre preparation [15], which had an equal TDF content in the final in vitro digesta. The other components of the cotyledons have thus not caused any further decrease in bile acid release. Therefore, the decrease in the apparent permeability rate constants can be primarily attributed to the dietary fibre contained in the cotyledon. In agreement with the rheological data (Section 3.3), the OHEP and CL fractions showed the highest decrease of the release rate, of about 65%, compared to the blank digestion. Therefore, increased bile acid excretion reported in vivo may be ascribed to impeded bile acid diffusion [11,12]. We assume that this is mainly due to the pectic polymers contained in the OHEP and CL fractions.

Lupin hulls also caused a retarded release of all primary bile acids, but it was significantly lower than that observed for the cotyledon. In agreement with its different composition and rheological behaviour, OHEP showed an extended retardation. The decrease in release rate was similar for hull, as well as for the HC and CL fractions. The retarded release for lupin hull can thus mainly be ascribed to these fractions. Since lignin did not show a significant increase in viscosity after in vitro digestion (Section 3.3.1), the reduced release rate can be explained by its adsorptive properties (Section 3.4.2). Cellulose is known to have a low bile acid binding capacity [60]. Accordingly, no significant impact on bile acid release was measurable. Although lupin hull is predominantly composed of cellulose, the hull had a stronger impact on bile acid release, which could be explained by the additional fibre components described in Section 3.2.

#### 3.4.2. Adsorptive Interactions

Based on the equilibrium concentrations (C_f_, Equation (1)), bile acid adsorption of fractionated CWM derived from lupin cotyledon and hull, as well as from the references, cellulose and lignin, were calculated with respect to dry matter concentration in the in vitro digesta. Conjugation of bile acids had no significant effect on the adsorption (two-way ANOVA, *p* = 0.5). Therefore, the data for glyco- and tauroconjugated cholic acids and chenodesoxycholic acids are given and summarized in Table 4.

Lignin showed the highest adsorption capacity, which was significant for cholic acids (1.29 µmol/100 mg DM) and chenodesoxycholic acids (4.13 µmol/100 mg DM, Table 4). Lupin cotyledon showed significant adsorption of chenodesoxycholic acids of 1.29 µmol/100 mg DM (Table 4). The higher adsorption of chenodesoxycholic acids compared to cholic acids observed for lupin cotyledon and lignin (Figure 6 and Table 4) is in line with the results of our previous study and supports the hypothesis of a hydrophobic linkage between bile acids and dietary fibres [15]. Due to the low lignin content of the cotyledons (0.37 g/100 g DM, Table 1), the adsorption observed for the cotyledon cannot be attributed to lignin. For lupin hull, fractionated hull CWM, and cellulose (Figure 6 and Table 4) no significant adsorption of primary bile acids was observed. Thus, the low proportion of lignin in lupin hull did not affect the adsorptive capacity of the hull.

**Figure 7:**
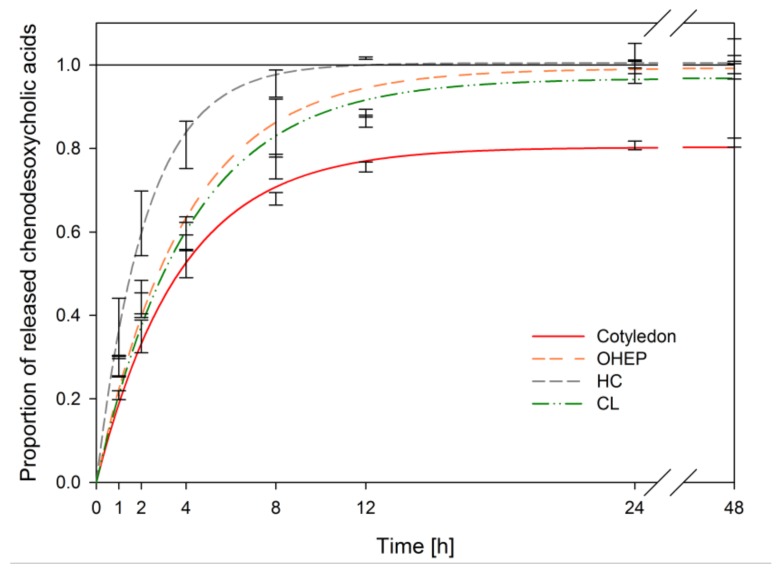
Release kinetics of chenodesoxycholic acids of in vitro digested fractionated dietary fibre (pectin-like fraction (OHEP), hemicellulose fraction (HC), cellulose-lignin fraction (CL)) derived from lupin cotyledon of *L. angustifoilus* L. Boregine (*n* = 3).

In Figure 7, the kinetics of chenodesoxycholic acid release of the whole cotyledon is compared to its cell wall polysaccharide fractions.

Bile acid release curves of in vitro digested cotyledon fractions (OHEP, HC, CL) deviated strongly from the release curve of the whole cotyledon (Figure 7). No significant adsorptive effects were measurable in dietary fibre fractions (Table 4). This contradicts our hypothesis that bile acids are adsorbed by insoluble dietary fibre polymers. As the results obtained after isolation and fractionation of cell walls disapproved a major role of lupin cell wall polysaccharides in bile acid adsorption, the adsorption observed for lupin cotyledon must be associated with other lupin cotyledon components.

The adsorptive capacity observed for lupin fibre preparation (1.74 µmol chenodesoxycholic acids/100 mg DM, Naumann et al. [15]) indicates that either the adsorption of lupin is caused by components associated with the cell wall polysaccharides or that the extraction process leads to an accumulation of components in the fibre fraction being able to adsorb bile acids. Besides the fibres (dietary fibre content 83.4 g/100 g DM), residual proteins (11.0 g/100 g DM) are the second main components of the fibre preparation derived after protein isolation. It has been described in several in vivo studies that lupin protein acts hypocholesterolemic [61,62,63], but the underlying mechanism is still not fully elucidated. An in vitro study performed by Yoshie-Stark and Wäsche [64] indicates a high bile acid binding capacity of protein isolates derived from white lupins. The bile acid adsorption observed for the cotyledon in our study may thus result from the adsorption of the lupin protein. Further studies are needed to investigate the adsorptive capacity of the lupin protein and to clarify the role of bile acid adsorption in cholesterol reduction.

Adsorptive capacities have also been suggested for dietary fibre sources with a low protein contents, such as described for a number of fruits by Kahlon and Smith [65]. The authors hypothesized that adsorption could be attributed to polyphenols. This hypothesis is supported by the adsorption we observed for lignin in our study. Unlike most dietary fibres, lignin is not a dietary fibre polysaccharide but a phenolic macromolecule [3]. It remains to be investigated to what extent polyphenols contribute to the adsorption of bile acids observed for dietary fibre preparations.

## 4. Conclusions

In our study, we investigated how bile acid interactions were influenced by the cell wall polysaccharides of lupin hull and cotyledon. Lupin hull mostly consisted of cellulosic polymers, which is in line with low bile acid retardation induced by viscosity. Bile acid adsorption observed for a lignin reference material was not evident in hull or cotyledon due to low lignification. Sequential extraction revealed that pectin-like substances are mainly responsible for viscosity in lupin cotyledon digesta. We suggest that the formation of entangled networks, causing predominantly elastic properties, majorly contributes to the increase of viscosity. Due to the characterization of components accountable for the viscous effects on bile acid reabsorption, our results could contribute to the elucidation of cholesterol-reducing mechanisms of lupin dietary fibres. In future studies, the effects of food processing on the composition and rheological properties of lupin dietary fibre should be addressed to ensure to transferability of the in vitro results to complex food matrices.

Our results indicate that bile acid adsorption of lupin cotyledon is not directly attributable to the cell wall polysaccharides. The contribution of dietary fibre polysaccharides to bile acid adsorption, as frequently reported for a variety of dietary fibre sources [21], should be considered more critically in future investigations. To improve clarity, the term binding should not be used for interactions between fibres and bile acids unless a molecular interaction is evident. Future studies should focus on adsorptive capacities of further lupin components, like proteins and phytochemicals, to identify relevant structures and mechanisms.

## Figures and Tables

**Table 1:** Chemical (dry matter (DM), ash, protein, fat, carbohydrates) and dietary fibre composition (total dietary fibre (TDF), soluble dietary fibre (SDF), insoluble dietary fibre (IDF), neutral detergent fibre (NDF), acid detergent fibre (ADF), and acid detergent lignin (ADL)) of the cotyledon and the hull of *L. angustifolius* L. Boregine (*n* = 3).

		Cotyledon	Hull
DM	[g/100 g]	88.13 ± 0.11	91.53 ± 0.02
ash	[g/100 g DM]	4.26 ± 0.14	2.60 ± 0.05
protein	[g/100 g DM]	40.13 ± 0.13	1.98 ± 0.04
fat	[g/100 g DM]	9.47 ± 0.07	<0.5
carbohydrates	[g/100 g DM]	8.38 ± 2.15	4.08 ± 0.76
starch	[g/100 g DM]	0.07 ± 0.02	0.03 ± 0.00
TDF	[g/100 g DM]	37.77 ± 2.14	91.31 ± 0.76
IDF	[g/100 g DM]	34.59 ± 0.96	90.10 ± 0.56
SDF	[g/100 g DM]	3.18 ± 1.91	1.21 ± 0.52
NDF	[g/100 g DM]	11.34 ± 1.52	82.59 ± 0.40
ADF	[g/100 g DM]	5.25 ± 0.30	74.81 ± 0.50
ADL	[g/100 g DM]	0.37 ± 0.09	1.95 ± 0.46

**Table 2:** Monosaccharide composition of cotyledon, hull and isolated pectin-like fraction (OHEP), hemicellulose fraction (HC), and cellulose-lignin fraction (CL) of *L. angustifolius* L. Boregine, expressed as a percentage of total polysaccharide sugars.

Sample	Arabinose	Rhamnose	Galactose	Glucose	Xylose	Mannose	Uronic Acids
Cotyledon	11.5	1.8	67.6	7.6	2.6	0.8	8.1
C_OHEP	11.3	1.6	73.2	1.1	2.0	0.3	10.5
C_HC	14.0	1.7	71.8	2.0	2.7	0.1	7.8
C_CL	10.6	1.2	46.9	24.8	3.5	0.7	12.3
Hull	8.3	0.4	1.8	59.1	15.7	2.6	12.1
H_OHEP	20.7	1.4	13.6	1.2	7.5	25.9	29.7
H_HC	2.9	0.4	4.5	9.9	71.0	5.3	6.1
H_CL	7.0	0.4	0.4	77.3	3.7	0.8	10.4

**Table 3:** Apparent permeability rate constants (k) of kinetic bile acids release analysis (glycocholic acid (GCA), taurocholic acid (TCA), glycochenodesoxycholic acid (GCDC), and taurochenodesoxycholic acid (TCDC)) of in vitro digesta containing fractionated dietary fibre (pectin-like fraction (OHEP), hemicellulose fraction (HC), cellulose-lignin fraction (CL)) derived from lupin cotyledon and hull of *L. angustifolius* L. Boregine, cellulose and lignin (*n* = 3).

Sample	k [h^−1^]			
	GCA	TCA	GCDC	TCDC
Cotyledon	0.48 ± 0.08 ^a,b^	0.44 ± 0.07 ^a,b,c^	0.28 ± 0.03 ^a^	0.26 ± 0.03 ^a^
C_OHEP	0.36 ± 0.03 ^a^	0.38 ± 0.04 ^a,b^	0.25 ± 0.02 ^a^	0.26 ± 0.03 ^a^
C_HC	0.58 ± 0.10 ^b,c^	0.57 ± 0.08 ^b,c,d^	0.47 ± 0.14 ^b^	0.45 ± 0.06 ^b^
C_CL	0.36 ± 0.06 ^a^	0.31 ± 0.05 ^a^	0.26 ± 0.04 ^a^	0.23 ± 0.04 ^a^
Hull	0.74 ± 0.04 ^c,d^	0.64 ± 0.04 ^c,d,e^	0.49 ± 0.03 ^b^	0.46 ± 0.02 ^b^
H_OHEP	0.39 ± 0.03 ^a^	0.34 ± 0.02 ^a^	0.28 ± 0.01 ^a^	0.26 ± 0.01 ^a^
H_HC	0.69 ± 0.05 ^c,d^	0.65 ± 0.06 ^d,e^	0.51 ± 0.02 ^b,c^	0.48 ± 0.02 ^b^
H_CL	0.85 ± 0.04 ^d,e^	0.74 ± 0.04 ^d,e,f^	0.62 ± 0.03 ^b,c,d^	0.59 ± 0.01 ^c,d^
Cellulose	0.92 ± 0.07 ^e,f^	0.82 ± 0.06 ^e,f^	0.65 ± 0.06 ^c,d^	0.63 ± 0.06 ^c,d^
Lignin	0.74 ± 0.04 ^c,d^	0.71 ± 0.05 ^d,e,f^	0.59 ± 0.03 ^b,c,d^	0.54 ± 0.03 ^b,c^
Blank	0.96 ± 0.04 ^f^	0.93 ± 0.02 ^f^	0.72 ± 0.03 ^d^	0.65 ± 0.01 ^d^

Along the column, different letters indicate significant differences on a *p* ≤ 0.05 level basis.

**Table 4:** Bile acid adsorption (given as sum of glyco- and tauroconjugated cholic acids and chenodesoxycholic acids) of in vitro digesta containing fractionated dietary fibre (pectin-like fraction (OHEP), hemicellulose fraction (HC), cellulose-lignin fraction (CL)) derived from lupin cotyledon and hull of *L. angustifolius* L. Boregine, cellulose and lignin (*n* = 3).

Sample	Bile Acid Adsorption [μmol/100 mg DM]	
	Cholic Acids	Chenodesoxycholic Acids
Cotyledon	0.16 ± 0.26 ^a,b^	1.29 ± 0.09 ^b^
C_OHEP	−0.02 ± 0.50 ^b^	0.12 ± 0.70 ^a^
C_HC	−0.03 ± 0.26 ^b^	−0.08 ± 0.19 ^a^
C_CL	0.19 ± 0.23 ^b^	0.50 ± 0.31 ^a,b^
Hull	0.22 ± 0.09 ^b^	0.02 ± 0.20 ^a^
H_OHEP	0.10 ± 0.19 ^b^	0.01 ± 0.26 ^a^
H_HC	0.27 ± 0.36 ^b^	0.29 ± 0.17 ^a,b^
H_CL	0.19 ± 0.68 ^b^	0.26 ± 0.79 ^a,b^
Cellulose	−0.05 ± 0.12 ^b^	−0.01 ± 0.25 ^a^
Lignin	1.29 ± 0.26 ^a^	4.13 ± 0.42 ^c^
Blank	0.00 ± 0.06 ^b^	−0.15 ± 0.04 ^a^

Along the column, different letters indicate significant differences on a *p* ≤ 0.05 level basis.

## Abbreviations

DM	dry matter
GCA	glycocholic acid
GCDC	glycochenodesoxycholic acid
TCA	taurocholic acid
TCDC	taurochenodesoxycholic acid
